# Somatic Mutations and the Risk of Undifferentiated Autoinflammatory Disease in MDS: An Under-Recognized but Prognostically Important Complication

**DOI:** 10.3389/fimmu.2021.610019

**Published:** 2021-02-19

**Authors:** Abdulla Watad, Mark Kacar, Nicola Luigi Bragazzi, Qiao Zhou, Miriam Jassam, Jan Taylor, Eve Roman, Alexandra Smith, Richard A. Jones, Howard Amital, Catherine Cargo, Dennis McGonagle, Sinisa Savic

**Affiliations:** ^1^National Institute for Health Research—Leeds Biomedical Research Centre and Leeds Institute of Rheumatic and Musculoskeletal Medicine (LIRMM), Wellcome Trust Brenner Building, St James's University Hospital, Leeds, United Kingdom; ^2^Department of Medicine B and Zabludowicz Center for Autoimmune Diseases, Sheba Medical Center, Tel-Hashomer, Ramat-Gan, Israel, Sackler Faculty of Medicine, Tel-Aviv University, Tel-Aviv, Israel; ^3^Department of Clinical Immunology and Allergy, St James's University Hospital, Leeds, United Kingdom; ^4^Laboratory for Industrial and Applied Mathematics (LIAM), Department of Mathematics and Statistics, York University, Toronto, ON, Canada; ^5^Department of Rheumatology & Immunology, Sichuan Academy of Medical Sciences & Sichuan Provincial People's Hospital, Chengdu, China; ^6^Department of Haematology, St James's University Hospital, Leeds, United Kingdom; ^7^Epidemiology & Cancer Statistics Group, Department of Health Sciences, University of York, York, United Kingdom; ^8^HMDS Department, Leeds Teaching Hospitals, Leeds, United Kingdom

**Keywords:** myelodysplatic syndrome, autoinflammation, molecular characterization, somatic mutations, undifferentiated autoinflammatory disease

## Abstract

**Objectives:** We theorized that myelodysplastic syndrome (MDS) with somatic mutations and karyotype abnormalities are associated with autoinflammation, and that the presence of autoinflammatory disease affected prognosis in MDS.

**Methods:** One hundred thirty-four MDS patients were assessed for the prevalence of autoinflammatory complications and its link with karyotypes and somatic mutation status. Autoinflammatory complications were described either as well-defined autoinflammatory diseases (AD) or undifferentiated “autoinflammatory disease” (UAD) (defined as CRP over 10.0 mg/L on five consecutive occasions, taken at separate times and not explained by infection). Several patient characteristics including demographic, clinical, laboratory, cytogenetics charts, and outcomes, were compared between different groups.

**Results:** Sixty-two (46.3%) patients had an autoinflammatory complication manifesting as arthralgia (43.5% *vs*. 23.6%, *p* = 0.0146), arthritis (30.6% *vs*. 15.3%, *p* = 0.0340), skin rash (27.4% *vs*. 12.5%, *p* = 0.0301), pleuritis (14.5% *vs*. 4.2%, *p* = 0.0371) and unexplained fever (27.4% *vs*. 0%, *p* < 0.0001). AD were found in 7.4% of MDS patients (with polymyalgia rheumatic being the most frequently one). Classical autoimmune diseases were found only in 4 MDS patients (3.0%). Transcription factor pathway mutations (*RUNX1, BCOR, WTI, TP53*) (OR 2.20 [95%CI 1.02–4.75], *p* = 0.0451) and abnormal karyotypes (OR 2.76 [95%CI 1.22–6.26], *p* = 0.0153) were associated with autoinflammatory complications. Acute leukaemic transformation was more frequent in MDS patients with autoinflammatory features than those without (27.4% *vs*. 9.7%, *p* = 0.0080).

**Conclusions:** Autoinflammatory complications are common in MDS. Somatic mutations of transcription factor pathways and abnormal karyotypes are associated with greater risk of autoinflammatory complications, which are themselves linked to malignant transformation and a worse prognosis.

## Introduction

Myelodysplastic syndrome (MDS) and chronic myelomonocytic leukemia (CMML) are clonal haemopoietic disorders characterized by ineffective and dysplastic haematopoiesis of innate immune lineage cells, resulting in cytopenias and, in some cases, progression to acute myeloid leukemia (AML) (1). Several cytokines are crucial to hematopoietic stem cell (HSC) maturation and differentiation, especially during infection and inflammation, including TNF, IL-6 and IL-1β (2). Indeed, MDS is associated with dysfunction of the Nod-Like Receptor Protein 3 (NLRP3) inflammasome and aberrant Toll-like Receptor (TLR) signaling, both of which lead to the induction of several pro-inflammatory cytokines (i.e., IL-1β) strongly linked to autoinflammatory disease states in general (3). These cytokines may also promote tumorigenesis by driving cell proliferation and migration in MDS–especially in those subjects with abnormal karyotypes (4). The formation of reactive oxygen species (ROS) and subsequent NF-κB mediated increase in cytokine production was theorized to be the point of convergence of various pathways involved in the pathogenesis of MDS with inflammation (5). Additional factors playing a role in driving the MDS phenotype are now attributed to bone marrow (BM) stromal cells which appear to confer a survival advantage to mutant clones that would not survive outside the context of inflammation (6, 7).

The term “autoinflammation” was coined in 1999 to describe non-infectious inflammatory states characterized by the absence of self-directed autoantibodies or T-cells (8). The recognition that autoinflammation was synonymous with innate immune-mediated pathology and the functional integration of innate and adaptive immunity resulted in the immunological disease continuum classification, which allows for the recognition of all non-infectious inflammatory disease (9, 10). It is now evident that cells of the myeloid lineage (such as neutrophils), and of the monocyte lineage (such as macrophages), are key cellular players in the genesis of innate immune-mediated or autoinflammatory disorders (11).

Several studies have linked autoimmune disease to MDS, reporting a higher prevalence of B- and T-cell mediated disorders such as hypothyroidism, autoimmune haemolytic anemia, idiopathic thrombocytopenic purpura, and rheumatoid arthritis (12). Of note, most studies did not make a clear distinction between pure autoimmune and autoinflammatory disorders (13, 14). Given the links between autoinflammation and MDS within myeloid lineage cells, we hypothesized that autoinflammatory disorders may be more common in MDS than autoimmune diseases (9).

MDS is associated with several cytogenetic and somatic mutations, as well as epigenetic alterations that may not only affect the cell cycle but may also influence myeloid cell inflammatory responses, either directly or indirectly (15). These MDS-associated mutations are also found in clonal hematopoiesis of indeterminate potential (CHIP), which is a premalignant bone-marrow state, often found in individuals with no apparent cytopenias, and typically preceding MDS and hematological malignacies (16). Even at this pre-MDS stage, CHIP has been linked to autoimmune diseases (like anti-neutrophil cytoplasmic antibody-associated vasculitis, ulcerative colitis or rheumatoid arthritis) in a number of studies (17–19), with this link being complex, multi-factorial and probably bidirectional (20). Genetic factors, age, inflammation, environmental stressors and other variables can contribute to clonal evolution (19). For instance, age-related somatic mutations in the HSC compartment that give a competitive survival advantage may lead to clonal hematopoiesis. This, as previously mentioned, represents a premalignant condition for MDS and for myeloid neoplasms including AML but is also a risk factor for cardiovascular disease and thromboembolism, both of which are linked to aberrant inflammatory reactions. From a functional perspective, loss of function mutations in epigenetic modifiers including *DNMT3A, TET2* and other genes may result in IL-1 and IL-6 and other pro-inflammatory cytokine dysregulation and hence to inflammation (20). To date, no study has explored the link between MDS-associated cytogenetic and somatic mutations, and autoinflammation/autoimmune complications. This study therefore investigated the hypothesis that autoinflammatory disease is common in MDS cohorts, further postulating that the association was stronger between autoinflammatory conditions and specific MDS-associated somatic mutations and karyotypic abnormalities.

## Materials and Methods

### Ethical Approval

The study protocol of the present investigation received ethical clearance from Leeds University, UK. This study was conducted in accordance with the ethical guidelines and principles of the 1964 Helsinki declaration and its subsequent amendments. The Hematological Malignancy Research Network (HMRN) has ethics approval (REC 04/01/1205/69) from Leeds West Research Ethics Committee.

### Data Source

This research was carried out on patients from the Yorkshire Hematological Malignancy Research Network (HMRN). The HMRN was established in 2004 to provide robust generalizable data to inform clinical practice and research (21). It comprises an ongoing population-based cohort of patients newly diagnosed by a single integrated haemato-pathology laboratory [Hematological Malignancy Diagnostic Service (HMDS)] covering a population of 3.6 million. The database includes prognostic factors and sequential treatment/response history; socio-demographic details are recorded to clinical trial standards.

### Patients

Any patient with a confirmed diagnosis of MDS or a myelodysplastic/myeloproliferative overlap syndrome between 2014 and 2017, at St. James's University Hospital in Leeds, was systematically recruited in the present retrospective study (*n* = 160). Of these samples, 134 had undergone targeted gene sequencing and formed the final cohort for analysis (see flowchart). Cytogenetic data was available on 111 patients. The following parameters were extracted from medical charts: age, gender, MDS subtype (according to the 2008 revised WHO classification), clinical symptoms/signs (unexplained fever, arthritis, arthralgia, skin rash, sore throat, oral ulcers, neurological and visual impairment), imaging findings (pericarditis, peritonitis, pleuritis), laboratory findings (leukocytosis [>12,000/mL], ferritin [>500 mg/L], anemia, neutropenia, lymphopenia, thrombocytopenia, presence of auto-antibodies, hypo- and hyperthyroidism), treatment received (erythropoietin/granulocyte-colony stimulating factor, hypomethylating agents, chemotherapy, biological therapy, bone marrow transplantation) and prognosis (transformation to acute leukemia, the prognostic risk categories based on the Revised International Prognostic Scoring System (IPSS-R), and mortality rate (with cases of death registered up to 20 of December, 2018).

### Definition of Autoinflammation

Established diagnosis of autoinflammatory disease (AD) as defined by the immunological disease continuum model (9). Examples include neutrophilic inflammatory disorders, diseases without associated autoantibodies and chronic inflammatory diseases without strong MHC class II associations. Several diseases including giant cell arteritis (GCA) and polymyalgia rheumatica (PMR) have clinical features that are intermediate between pure autoimmunity and pure autoinflammation, although the latter appears to be the predominant component in these disorders (9, 22, 23).

#### Undifferentiated Autoinflammatory Disease (UAD)

The UAD was defined by the presence of symptoms such as fevers, fatigue, arthralgia, myalgia [suggestive of autoinflammatory disorder (14)] in association with persistently elevated CRP over 10.0 mg/l. The CRP had to be elevated on five consecutive occasions taken at separate times and not explained by infection (normal chest x-ray, urine culture, blood culture and total body CT in some cases).

### Molecular Characterization

Genomic DNA was extracted from the bone marrow aspirate samples using the using the QIAamp DNA mini kit (QIAGEN, Manchester, UK) in accordance with protocols approved by an institutional review board. Targeted gene sequencing of 27 genes recurrently mutated in myeloid malignancies was performed on the MiSeq (Illumina, Chesterford, UK) (23). The following genes were assessed: transcription factors (*RUNX1, BCOR, WTI, TP53*), DNA methylation (*TET2, DNMT3A, IDH1, IDH2*), chromatin modification (*ASXL1, EZH2*), splicing (*SF3B1, SRSF2, U2AF1, ZRSR2*), signaling (*FLT3, NRAS, KRAS, CBL, cKIT, JAK2, MPL, CSF3R*), cohesion complex (*STAG2*) and others (*NPM1, SETBP1, CALR*). Panel design, validation and variant filtering criteria are those reported in our previous publication (24). The mean coverage of identified variants was 1514x (ranging from 52 to 5605x). Karyotypic abnormalities were categorized as per Schanz et al. (25).

### Statistical Analysis

Before commencing any statistical analysis, data were visually inspected for potential outliers. Continuous data were expressed as mean ± standard deviation, whereas categorical variables were computed as percentages. Univariate (chi-squared test, Student's *t*-test or its parametric version in case of violation of normality of data distribution) and multivariate analyses were conducted to identify differences between MDS patients with and without overall autoinflammation, well-defined autoinflammatory disease and UAS, and to shed light on the determinants of autoinflammation. Multivariate analyses were performed choosing as predictors those variables statistically significant at univariate analyses and adjusting for age, gender, class of risk and treatment received.

Kaplan-Meyer and Cox proportional-hazards regression survival curve analyses were performed to assess likelihood of survival among MDS patients with and without autoinflammation. Cox analysis was conducted correcting for age, gender, class of risk and treatment received.

For all statistical analyses, figures with *p*-values equal to or <0.05 were considered statistically significant. Statistical analysis was performed using the commercial software “Statistical Package for the Social Sciences” (IBM SPSS Statistics for Windows, version 24.0, IBM Corp., Armonk, NW, USA; released in 2016).

## Results

### MDS Study Population Characteristics

**Figure 1:**
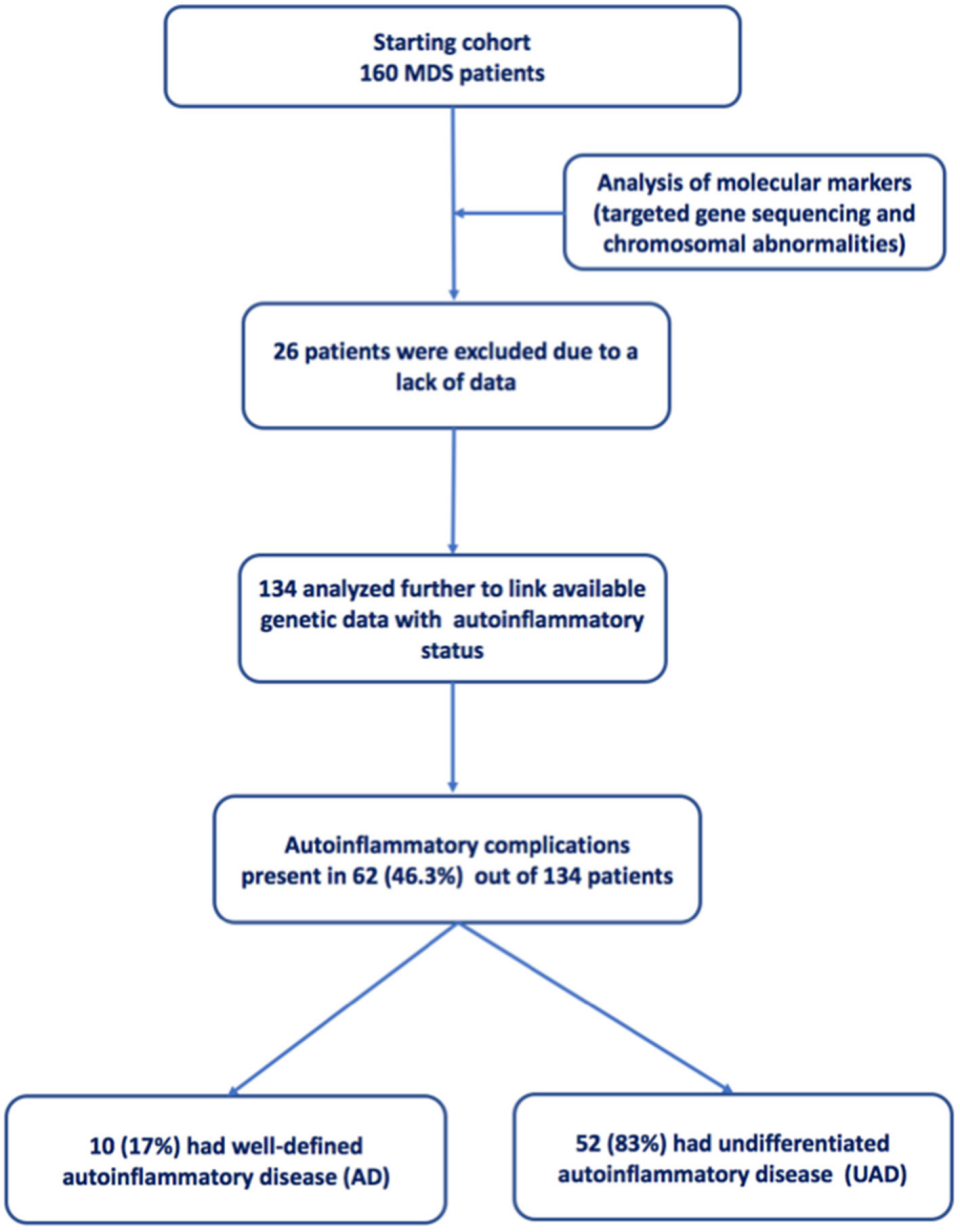
Flow chart describing the screening and selection of MDS patients.

A total of 160 patients were initially identified over the 3-year study period. Further, detailed analysis was performed on 134 patients who had complete set of cytogenetic and molecular investigations (Figure 1). All results reported below relate to this group. The average age was 75.2 ± 11.6 years (median 77.5 years), with the majority of subjects being male (*n* = 85, 63.4%) (Table 1).

**Table 1:** Major characteristics of the cohort recruited.

**Parameter**	**Overall cohort**
	**(*n* = 134)**
Age (*n* = 134)	75.16 ± 11.57; 77.5
Gender (male; *n* = 134)	85 (63.4%)
Symptoms and signs (*n* = 134)	
Arthralgia/arthritis	44 (32.8%)
Arthralgia	44 (32.8%)
Arthritis	30 (22.4%)
Skin rash	26 (19.4%)
Neurological symptoms	19 (14.2%)
Recurrent fever	17 (12.7%)
Oral ulcers	12 (9.0%)
Pleuritis	12 (9.0%)
Sore throat	6 (4.5%)
Visual impairments	5 (3.7%)
Pericarditis	2 (1.5%)
Peritonitis	1 (0.7%)
Laboratory examinations (*n* = 134)	
Anemia	104 (77.6%)
Thrombocytopenia	78 (58.2%)
Neutropenia	46 (34.3%)
Ferritin >500	36 (26.9%)
Lymphopenia	29 (21.6%)
Autoantibodies	18 (13.4%)
Hypothyroidism	17 (12.7%)
Leukocytosis >12K	17 (12.7%)
Hyperthyroidism	2 (1.5%)
Cytogenetics class (*n* = 111)	
Normal	63 (56.8%)
Other single or double independent clones	12 (10.8%)
Very complex	10 (9.0%)
+8	7 (6.3%)
−7	6 (5.4%)
Double including del(5q)	4 (3.6%)
del(5q)	3 (2.7%)
Complex	2 (1.8%)
–Y	2 (1.8%)
Double including −7/del(7q)	1 (0.9%)
Inv(3)/t(3q)/del(3q)	1 (0.9%)
Treatment (*n* = 134)	
Erythropoietin/G-CSF	35 (26.1%)
Hypomethylating agents	31 (23.1%)
Chemotherapy	12 (9.0%)
Bone marrow transplantation	11 (8.2%)
Hydroxyurea	8 (6.0%)
Clinical sub-type/category (*n* = 134)	
Refractory cytopenia with multilineage dysplasia	50 (37.3%)
Refractory anemia with excess blasts	36 (26.9%)
Chronic myelomonocytic leukemia	27 (20.1%)
Refractory anemia with ring sideroblasts	10 (7.5%)
Myelodysplastic/myeloproliferative neoplasm unclassified	4 (3.0%)
MDS with isolated del(5q)	3 (2.2%)
Atypical chronic myeloid leukemia	2 (1.5%)
MDS unclassifiable	1 (0.7%)
Refractory cytopenia with unilineage dysplasia	1 (0.7%)
Prognosis (*n* = 134)	
IPSS-R (*n* = 111)	
Very poor	10 (9.0%)
Poor	10 (9.0%)
Intermediate	19 (17.1%)
Good	70 (63.1%)
Very good	2 (1.8%)
Transformation to acute leukemia	24 (17.9%)
Death	78 (58.2%)

*CMML, Chronic myelomonocytic leukemia; MDS del5(q), MDS associated with partial chromosome 5 missing; MDS-U, MDS undefined; RAEB, Refractory anemia with excess blasts; RARS, Refractory anemia with ringed sideroblasts; RCMD, Refractory cytopenia with multilineage dysplasia; RCUD, Refractory cytopenia with unilineage dysplasia*.

Concerning clinical sub-type/category, most patients had refractory cytopenia with multi-lineage dysplasia (RCMD) (*n* = 50, 37.3%), followed by refractory anemia with excess blasts (RAEB) (*n* = 36, 26.9%), chronic myelomonocytic leukemia (CMML) (*n* = 27, 20.1%), and refractory anemia with ringed sideroblasts (RARS) (*n* = 10, 7.5%). More details regarding other clinical sub-type/category are reported in Table 1.

Regarding the treatment of MDS most patients received erythropoietin and/or G-CSF (*n* = 35, 26.1%), hypomethylating agents (*n* = 31, 23.1%), chemotherapy (*n* = 12, 9.0%) and hydroxyurea (*n* = 8, 6.0%), while bone marrow transplantation was required in 8.2% of cases (*n* = 11). The most frequent laboratory findings were anemia (*n* = 104, 77.6%), thrombocytopenia (*n* = 78, 58.2%), neutropenia (*n* = 46, 34.3%), increased levels of ferritin >500 (*n* = 36, 26.9%).

The most frequently reported inflammatory symptoms and signs in the overall study population were arthralgia (*n* = 44, 32.8%), arthritis (*n* = 30, 22.4%), skin rash (*n* = 26, 19.4%), neurological symptoms (*n* = 19, 14.2%), recurrent fever (*n* = 17, 12.7%), pleuritis (*n* = 12, 9.0%) and oral ulcers (*n* = 12, 9.0%) (Table 1). Overall, arthralgia and arthritis were jointly present in 44 subjects (32.8%), with 30 of them (68.2%) presenting both symptoms.

According to the MDS IPSS-R cytogenetic risk group stratification, 10 patients (9.0%) had a very poor prognosis, a further 10 (9.0%) a poor prognosis, and 19 (17.1%), 70 (63.1%), and 2 (1.8%) subjects respectively, had an intermediate, good or very good prognosis. Overall mortality rate was 58.2% (median follow-up 680 days) (Table 1).

### Comparison Between MDS Patients With and Without Autoinflammatory Complications

A total of 62 (46.3%) patients had either a well-defined AD or an UAD. These groups were younger than the MDS patients without any autoinflammatory complications (73.0 ± 12.6 years *vs*. 76.7 ± 11.1 years, borderline significant). The most frequent symptoms in AD and UAS groups collectively compared to patients without autoinflammatory complications included arthritis (*n* = 19, 30.6%, *vs. n* = 11, 15.3%, *p* = 0.0340), arthralgia (*n* = 27, 43.5%, *vs. n* = 17, 23.6%, *p* = 0.0146), skin rash (*n* = 17, 27.4%, *vs. n* = 9, 12.5%, *p* = 0.0301), pleuritis (*n* = 9, 14.5%, *vs. n* = 3, 4.2%, *p* = 0.0371) and unexplained fever (*n* = 17, 27.4% *vs. n* = 0, 0.0%, *p* < 0.0001) (Table 2).

**Table 2:** Differences between MDS patients with and without overall, well-defined (AD) and undifferentiated autoinflammatory disease (UAD).

**Parameter**	**MDS without autoinflammation**	**MDS/UAD**	**MDS/AD (*n* = 10)**	**MDS AD + UAD**	**Statistical significance**
	**(*n* = 72)**	**(*n* = 52)**		**(*n* = 62)**	
Age	76.74 ± 11.10; 78.5	72.98 ± 12.58; 76	75.10 ± 8.03; 79	73.22 ± 11.93; 77	NS
Gender (male)	48 (66.7%)	32 (61.5%)	5 (50.0%)	37 (59.7%)	NS
Symptoms					
Arthritis	11 (15.3%)	12 (23.1%)	7 (70.0%)	19 (30.6%)	0.0005
Arthralgia	17 (23.6%)	19 (36.5%)	8 (80.0%)	27 (43.5%)	0.0014
Skin rash	9 (12.5%)	14 (26.9%)	3 (30.0%)	17 (27.4%)	NS
Sore throat	2 (2.8%)	3 (5.8%)	1 (10.0%)	4 (6.5%)	NS
Neurological symptoms	10 (13.9%)	7 (13.5%)	2 (20.0%)	9 (14.5%)	NS
Visual impairments	3 (4.2%)	2 (3.8%)	0 (0.0%)	0 (0.0%)	NS
Pericarditis	1 (1.4%)	1 (1.9%)	0 (0.0%)	1 (1.6%)	NS
Peritonitis	0 (0.0%)	1 (1.9%)	0 (0.0%)	1 (1.6%)	NS
Pleuritis	3 (4.2%)	8 (15.4%)	1 (10.0%)	9 (14.5%)	NS
Oral ulcers	5 (6.9%)	6 (11.5%)	1 (10.0%)	7 (11.3%)	NS
Unexplained fever	0 (0.0%)	14 (26.9%)	3 (30.0%)	17 (27.4%)	<0.0001
Laboratory examinations					
Leukocytosis >12K	8 (11.1%)	7 (13.5%)	2 (20.0%)	9 (14.5%)	NS
Ferritin >500	12 (16.7%)	19 (36.5%)	5 (50.0%)	24 (38.7%)	0.011
Anemia	52 (72.2%)	44 (84.6%)	8 (80.0%)	52 (83.9%)	NS
Neutropenia	24 (33.3%)	20 (38.5%)	2 (20.0%)	22 (35.5%)	NS
Lymphopenia	17 (23.6%)	11 (21.2%)	1 (10.0%)	12 (19.4%)	NS
Thrombocytopenia	41 (56.9%)	33 (63.5%)	4 (40.0%)	37 (59.7%)	NS
Autoantibodies	9 (12.5%)	7 (13.5%)	2 (20.0%)	9 (14.5%)	NS
Hypothyroidism	8 (11.1%)	6 (11.5%)	3 (30.0%)	9 (14.5%)	NS
Hyperthyroidism	1 (1.4%)	1 (1.9%)	0 (0.0%)	1 (1.6%)	NS
Treatment					
G-CSF	15 (20.8%)	20 (38.5%)	0 (0.0%)	20 (32.2%)	0.013
Hypomethylating agents	9 (12.5%)	20 (38.5%)	2 (20.0%)	22 (35.5%)	0.0032
Chemotherapy	4 (5.6%)	7 (13.5%)	1 (10.0%)	8 (12.9%)	NS
Hydroxyurea	3 (4.2%)	5 (9.6%)	0 (0.0%)	5 (8.1%)	NS
Bone marrow transplantation	4 (5.6%)	7 (13.5%)	0 (0.0%)	7 (11.3%)	NS
Clinical sub-type/category					NS
ACML	0 (0.0%)	2 (3.8%)	0 (0.0%)	2 (3.2%)	
CML	11 (15.3%)	14 (26.9%)	2 (20.0%)	16 (25.8%)	
MDS unclassifiable	1 (1.4%)	0 (0.0%)	0 (0.0%)	0 (0.0%)	
MDS with isolated del(5q) MMNU	2 (2.8%)	1 (1.9%)	0 (0.0%)	1 (1.6%)	
MMNU	4 (5.6%)	0 (0.0%)	0 (0.0%)	0 (0.0%)	
RAEB	16 (22.2%)	18 (34.6%)	2 (20.0%)	20 (32.2%)	
RARS	9 (12.5%)	1 (1.9%)	0 (0.0%)	1 (1.6%)	
RCMD	28 (38.9%)	16 (30.8%)	6 (60.0%)	22 (35.5%)	
RCUD	1 (1.4%)	0 (0.0%)	0 (0.0%)	0 (0.0%)	
Prognosis					
Transformation to acute leukemia	7 (9.7%)	15 (28.8%)	2 (20.0%)	17 (27.4%)	0.023
Death	34 (47.2%)	38 (73.1%)	6 (60.0%)	44 (71.0%)	0.0157

*CMML, Chronic myelomonocytic leukemia; MDS del5(q), MDS associated with partial chromosome 5 missing; MDS-U, MDS undifferentiated; RAEB, Refractory anemia with excess blasts; RARS, Refractory anemia with ringed sideroblasts; RCMD, Refractory cytopenia with multilineage dysplasia; RCUD, Refractory cytopenia with unilineage dysplasia*.

### Well-Defined Autoinflammatory Diagnoses-AD Group

**Figure 2:**
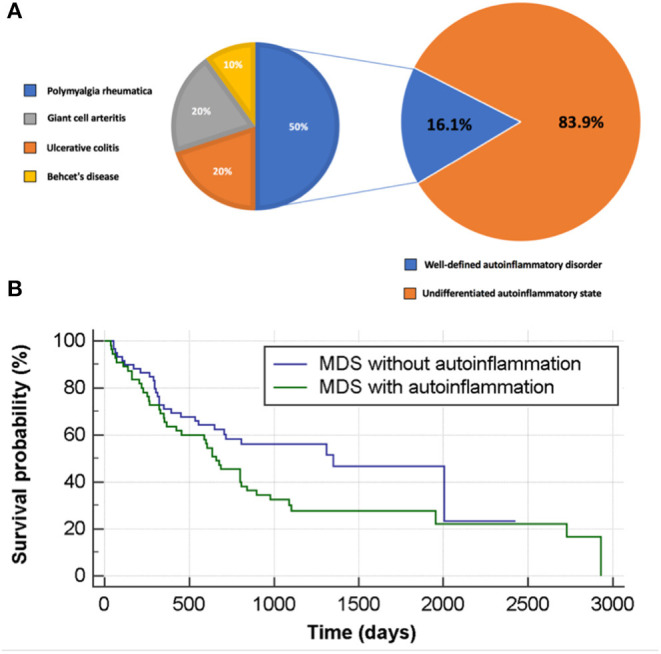
Autoinflammation in patients with MDS and its impact on survival rate. **(A)** Autoinflammatory state stratified to well-defined and undifferentiated autoinflammatory disorder in MDS patients. **(B)** Survival analysis for MDS patients with and without autoinflammation.

The definition of autoinflammations *vs*. autoimmune conditions was made according to the unified immunological disease classification proposed by our group in 2006 (9). A well-defined diagnosis of autoinflammatory disease was found for 10 patients (16.1% of MDS patients with autoinflammation). As depicted in Figure 2A, these were five cases of PMR, two cases of GCA, two cases of ulcerative colitis and one case of Behçet's disease (BD). All diagnoses were made after the MDS had been diagnosed. Further details regarding this patient group are shown in Supplementary Table 1. Some patients had two or more overlapping diagnoses, and few had additional inflammatory symptoms, which are atypical for their primary AD diagnosis. In addition several patients had difficult to treat AD recurring either prolonged courses of corticosteroids or additional treatments such as anakinra (patients AD10 in the Supplementary Table 1).

### Undifferentiated Autoinflammatory Disease (UAD)

Fifty-two patients had an UAD in the absence of a well-defined disease diagnosis. Among these, 26.9% had recurrent fever, 36.5% had arthralgia, 26.9% had skin rash, 23.1% had arthritis, 15.4% of had pleuritis and 1.9% had peritonitis. Of the UAD group, 69.2, 51.9, and 28.8%, respectively, had at least one, two and three clinical autoinflammatory features.

### Autoimmune Diagnoses

A diagnosis of autoimmune disorder was reported much less frequently than autoinflammatory complications, with former being present in only 4 MDS patients (3.0%). This included two cases of autoimmune thyroid disorder, one case of granulomatosis with polyangitis with c-ANCA positivity, and one case of autoimmune haemolytic anemia.

### The Role of Distinctive Karyotypes in Autoinflammatory State in AD and UAD

Karyotype abnormalities were found in 48 patients (43.2% of the entire sample). With respect to individual abnormalities, deletion of chromosome 5 (−5) was detected in seven patients (6.3%) whereas involvement of chromosome 7 (7) (monosomy 7/del7q) was found in further seven subjects (6.3%). In another seven patients (6.3%), (+8) was reported.

In univariate analysis (Table 3), any karyotype abnormality was found to be associated with autoinflammatory complications (*p* = 0.0069). However, at the level of specific categories of karyotype abnormality, no association was found with autoinflammation. In the *post-hoc* analysis, karyotype abnormality was statistically associated with UAD (*p* = 0.0127) (Table 3), yet no association was found with AD. Furthermore, in terms of IPSS-R cytogenetic risk stratification, univariate analysis demonstrated that good/very good risk groups were less associated with autoinflammatory status compared to those with higher risk groups (*p* = 0.0176), both in well-defined patients (*p* = 0.0035, *p* = 0.0028 for trend) and UAS patients (*p* = 0.0299, *p* = 0.0030 for trend). Furthermore, MDS patients with complex and very complex karyotype abnormalities had a significantly higher rate of autoinflammation complication than those with non-complex (83.3% *vs*. 76.8%, *p* = 0.0062).

**Table 3:** Cytogenetic characteristics of patients in relation to their autoinflammatory state.

**Parameter**	**MDS without autoinflammation (*n* = 58)**	**MDS/UAD(*n* = 45)**	**MDS/AD (*n* = 8)**	**MDS AD + UAD(*n* = 53)**	**Statistical significance**
Karyotype					NS
+8	4 (6.9%)	3 (6.7%)	0 (0.0%)	3 (5.7%)	
−7	1 (1.7%)	3 (6.7%)	2 (25.0%)	5 (9.4%)	
Double including del(5q)	3 (5.2%)	4 (8.9%)	0 (0.0%)	4 (7.5%)	
Double including−7/del(7q)	1 (1.7%)	0 (0.0%)	0 (0.0%)	0 (0.0%)	
Inv(3)/t(3q)/del(3q)	0 (0.0%)	1 (2.2%)	0 (0.0%)	1 (1.9%)	
Other single or double	5 (8.6%)	6 (13.3%)	1 (12.5%)	7 (13.2%)	
independent clones					
Complex	0 (0.0%)	1 (2.2%)	1 (12.5%)	2 (3.8%)	
Very complex	2 (3.4%)	7 (15.6%)	1 (12.5%)	8 (15.1%)	
Abnormal karyotype (versus normal)	18 (31.0%)	25 (55.6%)	5 (62.5%)	30 (56.6%)	0.0069 (comparing the three groups), 0.0127 (autoinflammaton *vs*. without autoinflammation)
Complex and very complex karyotype (versus non-complex)	2 (3.4%)	8 (17.8%)	2 (25.0%)	10 (18.9%)	0.0322 (comparing the three groups), 0.0062 (autoinflammaton *vs*. without autoinflammation)
MDS IPSS-R cytogenetic risk group					0.0176 (comparing the three groups), well-defined *vs*. without (*p* = 0.0035) and UAS *vs*. without (*p* = 0.0299)
Very poor	2 (3.4%)	7 (15.6%)	1 (12.5%)	8 (15.1%)	
Poor	2 (3.4%)	5 (11.1%)	3 (37.5%)	8 (15.1%)	
Intermediate	9 (15.5%)	9 (20.0%)	1 (12.5%)	10 (18.9%)	
Good	43 (74.1%)	24 (53.3%)	3 (37.5%)	27 (50.9%)	
Very good	2 (3.4%)	0 (0.0%)	0 (0.0%)	0 (0.0%)	

In multivariate analysis overall karyotype abnormalities were associated with overall autoinflammation (either AD or UAD) (OR 2.76 [95%CI 1.22–6.26], *p* = 0.0153). At the *post-hoc* analyses, overall karyotype abnormality was found to be associated with the UAD (OR 2.68 [95%CI 1.14–6.29], *p* = 0.0239), but not with AD. In terms of IPSS-R risk group stratification, good/very good risk groups were less associated with overall autoinflammation compared to those with higher risk (0.16 [95%CI 0.03–0.82], *p* = 0.0282) according to the IPSS-R scoring system. At the *post-hoc* analyses, patients with a good/very good prognosis according to the IPSS-R scoring system, were less associated with UAD (0.16 [95%CI 0.03–0.84], *p* = 0.0300).

### The Role of Somatic Mutations in Overall Autoinflammatory States

At least one somatic mutation was found in 91.9% of MDS patients. At the pathway level, somatic mutations involving the splicing, DNA methylation and transcription factors pathways were found in 27 (43.5%), 29 (46.8%), and 24 (38.7%) MDS cases respectively, whereas mutations of the chromatin modification pathway were involved in 21 patients (33.9%). Stratifying according to the autoinflammatory complications, the transcription factor pathway was mutated in 16 (22.2%) MDS patients without autoinflammation (AD + UAD), 21 (40.4%) with UAD, and 3 (30.0%) with AD (*p* = 0.0927).

At the specific gene mutation level, TET2, ASXL1, SRSF2, and SF3B1 were the most commonly reported in 36.6, 29.9, 20.1, and 19.5% in the overall MDS population, respectively. NRAS mutations was 1.4% in MDS patients without autoinflammatory complications, 21.2% in those with UAD, and 20.0% in those with AD (*p* = 0.0011). Also TP53 mutations were significantly higher in MDS patients with autoinflammation than those without (19.4 vs. 6.9%, *p* = 0.032). Further details are reported in Supplementary Table 2.

In univariate logistic regression analysis (Table 4), mutations affecting the transcription factors pathway were found to be predictive of autoinflammatory complications (OR 2.20 [95%CI 1.02–4.75], *p* = 0.0451). Stratifying according to autoinflammatory subtypes, mutations affecting the transcription factors pathway were associated with the risk of having UAD (OR 2.34 [95%CI 1.05–5.20], *p* = 0.0372). However, no statistically significant associations were found for AD, perhaps due to the small sample size. At a full multivariate logistic regression analysis, no predictor achieved statistical significance (Supplementary Tables 2–4).

**Table 4:** Genes mutations and karyotype abnormalities as predictors of overall, well-defined (AD) or undifferentiated autoinflammation (UAD) at the univariate logistic regression analysis.

**Parameter**	**AD** **+** **UAD**		**UAD**		**AD**	
	**OR [95%CI]**	**Statistical**	**OR [95%CI]**	**Statistical**	**OR [95%CI]**	**Statistical**
		**significance**		**significance**		**significance**
**GENE MUTATION**						
All genes mutated	0.92 [95%CI 0.24–3.51]	NS	1.02 [95%CI 0.25–4.23]	NS	0.70 [95%CI 0.06–8.11]	NS
DNA methylation pathway	0.97 [95%CI 0.46–2.04]	NS	0.96 [95%CI 0.43–2.14]	NS	1.09 [95%CI 0.26–4.58]	NS
Chromatin modification pathway	0.98 [95%CI 0.42–2.27]	NS	0.94 [95%CI 0.38–2.33]	NS	0.93 [95%CI 0.20–4.35]	NS
Transcription factors pathway	2.20 [95%CI 1.02–4.75]	0.0451	2.34 [95%CI 1.05–5.20]	0.0372	1.60 [95%CI 0.36–7.15]	NS
Splicing pathway	0.72 [95%CI 0.35–1.50]	NS	0.82 [95%CI 0.38–1.78]	NS	0.34 [95%CI 0.08–1.48]	NS
Cohesin complex pathway	1.59 [95%CI 0.44–5.78]	NS	2.01 [95%CI 0.54–7.54]	NS	–	–
Signaling pathway	1.65 [95%CI 0.65–4.22]	NS	1.77 [95%CI 0.67–4.69]	NS	0.96 [95%CI 0.15–6.11]	NS
Others	0.70 [95%CI 0.16–3.07]	NS	0.82 [95%CI 0.19–3.68]	NS	–	–
Number of mutations	1.26 [95%CI 0.95–1.67]	NS	1.34 [95%CI 1.00–1.80]	NS	0.91 [95%CI 0.49–1.67]	NS
**KARYOTYPE**						
Abnormal karyotype	OR 2.76 [95%CI 1.22–6.26]	0.0153	OR 2.68 [95%CI 1.14–6.29]	0.0239	3.67 [95% 0.72–18.64]	NS
**MDS IPSS-R CYTOGENETIC RISK GROUP**						
Poor *vs*. very poor	1.10 [95%CI 0.12–10.06]	NS	0.81 [95%CI 0.08–7.97]	NS	3.38 [95%CI 0.16–70.58]	NS
Intermediate *vs*. very poor	0.27 [95%CI 0.04–1.63]	NS	0.27 [95%CI 0.04–1.71]	NS	0.18 [95%CI 0.01–4.77]	NS
Good and very good *vs*. very poor	0.16 [95%CI 0.03–0.82]	0.0282	0.16 [95%CI 0.03–0.84]	0.0300	0.14 [95%CI 0.01–2.16]	NS

*CMML, Chronic myelomonocytic leukemia; MDS del5(q), MDS associated with partial chromosome 5 missing; MDS-U, MDS undifferentiated; RAEB, Refractory anemia with excess blasts; RARS, Refractory anemia with ringed sideroblasts; RCMD, Refractory cytopenia with multilineage dysplasia; RCUD, Refractory cytopenia with unilineage dysplasia*.

### The Role of Somatic Mutations and Pathways in Survival

At the KM analysis, *BCOR* (Chi-squared 7.38, *p* = 0.0066), *CBL* (Chi-squared 4.27, *p* = 0.0388), *SRSF2* (Chi-squared 4.17, *p* = 0.0411), and *TET2* (Chi-squared 10.54, *p* = 0.0012) were found to be associated with survival. A mutation affecting at least one of the genes associated with poor survival (namely, *BCOR, CBL, SRSF2*, and *TET2*) was highly associated with reduced survival median (Chi-squared 13.78, *p* = 0.0002): this remained significant, after adjusting for age, gender, treatment received and class of risk (HR 2.40 [95%CI 1.08–5.35], *p* = 0.0318) at the Cox analysis (Supplementary Figure 1).

Concerning the pathways, only DNA methylation (Chi-squared 3.95, *p* = 0.0470) impacted survival. In terms of number of gene mutations, an association with survival could be found (Chi-squared 14.33, *p* = 0.0136), whilst the presence of at least one gene mutated, regardless of its type, did not impact survival (Chi-squared 2.64, *p* = 0.1045) (Supplementary Figure 2). None of these variables remained significant after accounting for age, gender, treatment received and class of risk, at the Cox analysis.

### The Risk of Transformation and Mortality in MDS Patients With and Without an Autoinflammatory Complications

MDS patients with autoinflammatory diseases (AD and UAD) were significantly more likely to develop acute leukaemic transformation compared to non-complicated MDS (*n* = 17, 27.4%, *vs. n* = 7, 9.7%, *p* = 0.0080). Furthermore, the former group had a higher mortality rate (*n* = 44, 71.0%, *vs. n* = 34, 47.2%, *p* = 0.0056). No statistically significant differences could be found in terms of gender distribution, disease sub-type/category or IPSS-R (Table 2). Similar trends were seen for AD and UAD, with a transformation rate to acute leukemia of 20.0 and 28.8%, respectively. Likewise, the mortality rates for AD and UAD were 60.0 and 73.1%, respectively during the study period (Table 2). MDS patients without autoinflammatory complications had a mortality rate during the study period of 47.2% (at the Kaplan-Meyer analysis, chi-squared = 3.95, *p* = 0.0468, with a hazard ratio of survival of 1.62 [95%CI 1.01–2.62]; Figure 2B). This result holds also when adjusting for the class of risk (which was associated to survival, Chi-squared 37.08, *p* < 0.0001, Supplementary Figure 3) with a (HR of 1.90 [95%CI 1.00–3.62], *p* = 0.0500). However, at a full multivariate Cox analysis, adjusting for age, gender, class of risk and treatment received, autoinflammatory status failed to achieve statistical significance (HR 1.73 [95%CI 0.88–3.39], *p* = 0.1127).

## Discussion

Although the clinical spectrum of self-directed inflammation is highly heterogeneous it can be defined as two main types, one encompassing conditions with a primarily autoimmune component, and one with a primarily autoinflammatory or innate immune-mediated component (9). Diseases of adaptive immunity associated with aberrant B and T cell function including rheumatoid arthritis (RA), Sjogren's syndrome and coeliac disease, have been linked to lymphoma development (26). In this work, we show that the MDS disorders are even more strongly associated with diseases of innate immunity or autoinflammatory conditions. This is unsurprising given that disorders within the MDS spectrum are collectively linked to dysfunction of myeloid cells, which are key players in the innate immune system.

Many patients with MDS have non-specific symptoms and report fevers and elevated CRP that cannot be attributed to infection; we termed this group UAD. In our study the rate of overall autoinflammatory complications was very high and encompassed nearly half of the patients; most of these had an UAD. Our designation of such a category was supported by the finding that karyotype abnormalities were significantly higher in patients with autoinflammation than those without. It appears that an increased burden of somatic mutations correlates positively with a greater degree of innate immune cell dysregulation, a finding which is also prognostically relevant.

A unique feature of our study was the linking of MDS-related autoinflammation to karyotype abnormalities and somatic mutations. A number of studies have previously reported that autoimmune/autoinflammatory conditions are more common in MDS than in the general population, although these immune disorders were not well-delineated. The range of autoimmune/autoinflammatory disorders in patients with MDS is highly variable with an overall prevalence of 7–30% (14).

Several plausible mechanisms might explain the high prevalence of autoinflammation in MDS patients. Firstly, somatic mutations leading to clonal expansion have been reported to be a common occurrence during human aging. These mutations may affect different pathways such as signaling and DNA transcription pathways and therefore to lead to abnormal function of myeloid cells, with an increased secretion of various cytokines such as IL-1 and IL-6 (27). It has been reported that activation of the NLRP3 inflammasome in hematopoietic stem/progenitor cells is the critical convergent step in MDS development, with consequent clonal expansion and pyroptotic cell death through caspase-1 activation (3). The inflammasome is an intracellular multiprotein oligomer complex responsible for detecting pathogenic microorganisms and sterile stressors whose presence leads to the activation of pro-inflammatory cytokines IL-1 β and IL-18 (28). Recently, the new term “clonal haematopoiesis of indeterminate potential” (CHIP) has been introduced to describe acquisition of somatic mutations that drive clonal expansion in the absence of cytopenias and dysplastic haematopoiesis (16). Subsequently, it has been shown that the presence of CHIP in peripheral-blood cells is linked to nearly a doubling in the risk of ischaemic heart disease in humans and with accelerated atherosclerosis in mice; a condition already known as an inflammatory condition and predominantly driven by myeloid cells (29).

The main finding of our study is that abnormal karyotypes (of any subtype) and somatic mutations involving the transcription pathways, are associated with an increased incidence of autoinflammation, in particular of the UAD subtype. Furthermore, at the specific gene level, we found that NRAS and TP53 mutations are linked with increased risk of having autoinflammation. Interestingly, mutations in NRAS have been linked to causing selective immune abnormalities by increasing the RAF/MEK/ERK signaling, leading to the reduction of Bim, a proapoptotic protein and attenuates intrinsic, non-receptor-mediated mitochondrial apoptosis (30). Furthermore, TP53 has been reported to play an important role in innate immune responses through downregulating STAT-1 and therefore it's mutation may lead to dysregulated innate immunity and eventually, autoinflammation (31). Moreover, TP53 deficiency in animal models accelerated inflammatory arthritis (32).

With respect to the role of distinctive karyotypes, we found that patients in the “very good” and “good” risk groups (according to the IPSS cytogenetic classification) were at lower risk of presenting with autoinflammatory complications. In our cohort, patients with 5q deletion had a lower rate of autoinflammation compared to the overall MDS patients (28 *vs*. 46%, respectively) and were all diagnosed with UAD rather than AD.

A recent study has shown that autoimmune diseases are associated with distinctive karyotypes in patients with MDS, such as 5q deletion in neutrophilic dermatosis and trisomy 8 in Behcet's disease (33). Del(5q) is also associated with haploinsufficiency in Rps14, resulting in increased S100A8/9 (calprotectin) expression leading to myeloid-derived suppressor cell induction and subsequent reduction in antitumor immunity (34).

We found that MDS patients with autoinflammation have a worse prognosis than those without—a finding most probably related to the higher rate of acute leukaemic transformation in these subjects. The latter is probably attributed to the fact that there is a higher rate of autoinflammation complications among patients with “poor and very poor” than those with “very good and good” cytogenetic profile, known to have an important role in the risk of progression to acute myeloid leukemia. The impact of immune diseases on overall survival in MDS has been addressed by several studies of different designs, and showing conflicting findings. Similar to our results, Lee et al. (33) have shown that autoinflammatory disorders are associated with a 1.8-fold increase in mortality (95% CI 1.033–3.093; *P* = 0.038). By contrast, Komrokji et al. (35) reported that MDS patients with autoimmune diseases have better overall survival and lower risk of acute leukaemic transformation. Another study has shown that the overall survival was better in the group with autoimmune diseases (10.3 ± 0.6 [IC95% 6.2–12.9] vs. 4.8 ± 1.1 years) (36). Other studies found no significant impact of MDS-associated autoimmunity/autoinflammation on MDS patient survival (37, 38). The conflicting results of these studies might be attributed to the investigation of multiple variables, such as the specific type of autoinflammatory/autoimmune disorder, the subtype of MDS involved (and the karyotype abnormalities being observed), as well as the therapy these patients received. The main limitations of our study were its single-center nature and the lack of insight into the effect of MDS therapy on autoinflammation, due to the broad spectrum of therapeutic agents used in relation to the sample size of the study. Furthermore, GCA might be regarded as autoimmune and not autoinflammatory due to its known MHC-II associations. However, GCA is characterized by several ‘autoinflammatory' traits; namely the absence of associated autoantibodies and the characteristic inflammation of target tissues in the absence of lymphoid organ involvement (39).

We found that mutations affecting *BCOR, CBL, SRSF2*, and *TET2* were associated with reduced survival. Our results are in line with the existing scholarly literature. For instance, it has been found that some *BCOR* mutations impacted survival (40, 41). *CBL* mutations conferred as well reduced survival, with a 3-year survival rate of 27% (42), whereas *SRSF2* mutations are associated with aggressive MDS and poor survival (43). At the pathway level, genes involved in the DNA methylation pathway correlated with poor survival, replicating the findings of the literature (44–46).

Our study has a number of strengths, including the large, homogenous single-center series of patients. On the other hand, it suffers from some limitations, that should be properly acknowledged. The major shortcoming is represented by the retrospective study design and the utilization of clinical parameters. Future studies, while replicating our findings, should use also non-clinical parameters, like erythrocyte sedimentation rate (ESR), lymphocyte subsets, levels of IL-1B, TNF and other cytokines, to better provide more detailed biological and functional information. Moreover, some categories, such as CMML and other MPN, should have been analyzed separately from MDS population as per WHO guidelines, but this would have profoundly impacted the power of the statistical analyses performed. As such, these categories were grouped together in the current study. Future high-quality investigations should rely on larger sample sizes, to be able to carry out stratifications and sub-group analyses. In addition future studies will need to include new genetic mutations associated with MDS. For example this study did not include *UBA1*, which is a gene encoding for a critical E1 ligase. Somatic mutations in UBA1 have recently been linked with a severe autoinflammatory disorder called VEXAS (vacuoles, E1 enzyme, X-linked, autoinflammatory, somatic) where number of patients also have overlapping MDS and cytopenias (47).

In conclusion, autoinflammatory complication prevalence is higher in MDS patients than expected, especially within the UAS subtype. Patients with such complications might present to various specialist including, internal medicine physicians, geriatricians, immunologists and rheumatologists, who should be cognizant of this and should always look for MDS in elderly patients who develops systemic inflammatory manifestations. Patients with AD might have additional symptoms that might be related to their inflammatory MDS. Abnormal karyotypes and somatic mutations, especially those affecting the transcription factor pathways, are associated with an increased risk of autoinflammation. Furthermore, autoinflammation is linked to a worse prognosis which may be due to the higher risk of malignant transformation. The latter observation may have implications for the future use of selective NLRP3 inhibitors or other inflammasome inhibitors in this subgroup of patients. Further research is needed to ascertain whether prognosis can be improved using an approach based on the modulation of innate immunity.

## Data Availability Statement

The original contributions presented in the study are included in the article/Supplementary Material, further inquiries can be directed to the corresponding author/s.

## Ethics Statement

The studies involving human participants were reviewed and approved by The Hematological Malignancy Research Network (HMRN) has ethics approval (REC 04/01/1205/69) from Leeds West Research Ethics Committee. Written informed consent for participation was not required for this study in accordance with the national legislation and the institutional requirements.

## Author Contributions

AW, MK, QZ, MJ, JT, and ER collected the data. NB, JT, ER, AS, RJ, CC, and HA analyzed the data. SS, DM, and CC designed the research. AW, MK, DM, CC, and SS wrote the first draft of the manuscript. SS and DM funded the research. All authors approved the manuscript.

## Conflict of Interest

SS declares that he has received a travel grant and honoraria from SOBI and Novartis. The remaining authors declare that the research was conducted in the absence of any commercial or financial relationships that could be construed as a potential conflict of interest.

## Supplementary Material

Supplementary Figure 1 Survival curve analyses stratified according to gene mutation (green: mutation; blue: no mutation).

Supplementary Figure 2 Survival curve analysis based on gene mutations affecting a specific pathway.

Supplementary Figure 3 Overall survival stratified according to risk class (blue: very poor, light green: poor, orange: intermediate, green: good and violet: very good).

Supplementary Table 1 Established diagnoses of autoinflammatory disease (AD) and additional inflammatory features.

Supplementary Table 2 Multivariate logistic regression analysis of the determinants associated with overall auto-inflammation.

Supplementary Table 3 Multivariate logistic regression analysis of the determinants associated with undifferentiated autoinflammation.

Supplementary Table 4 Multivariate logistic regression analysis of the determinants associated with well-defined autoinflammation.
