# Supplementary figures and images for: Somatic Mutations and the Risk of Undifferentiated Autoinflammatory Disease in MDS: An Under-Recognized but Prognostically Important Complication
Source: Front Immunol. 2021 Feb 19;12:610019. doi: 10.3389/fimmu.2021.610019 (PMC7933213; doi:10.3389/fimmu.2021.610019)

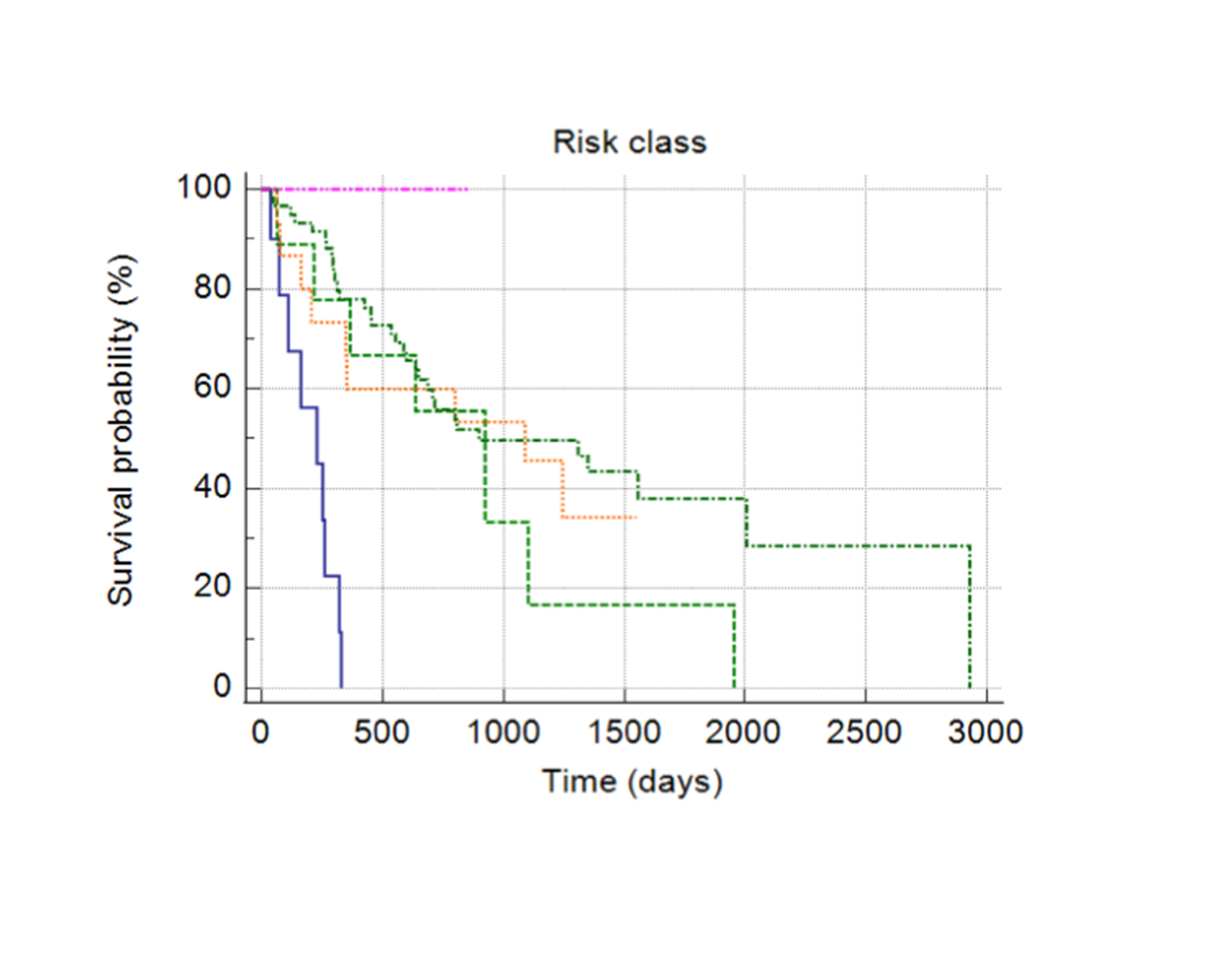

Supplement: Supplementary Figure 1 — Survival curve analyses stratified according to gene mutation (green: mutation; blue: no mutation). [file Image_1.TIFF]

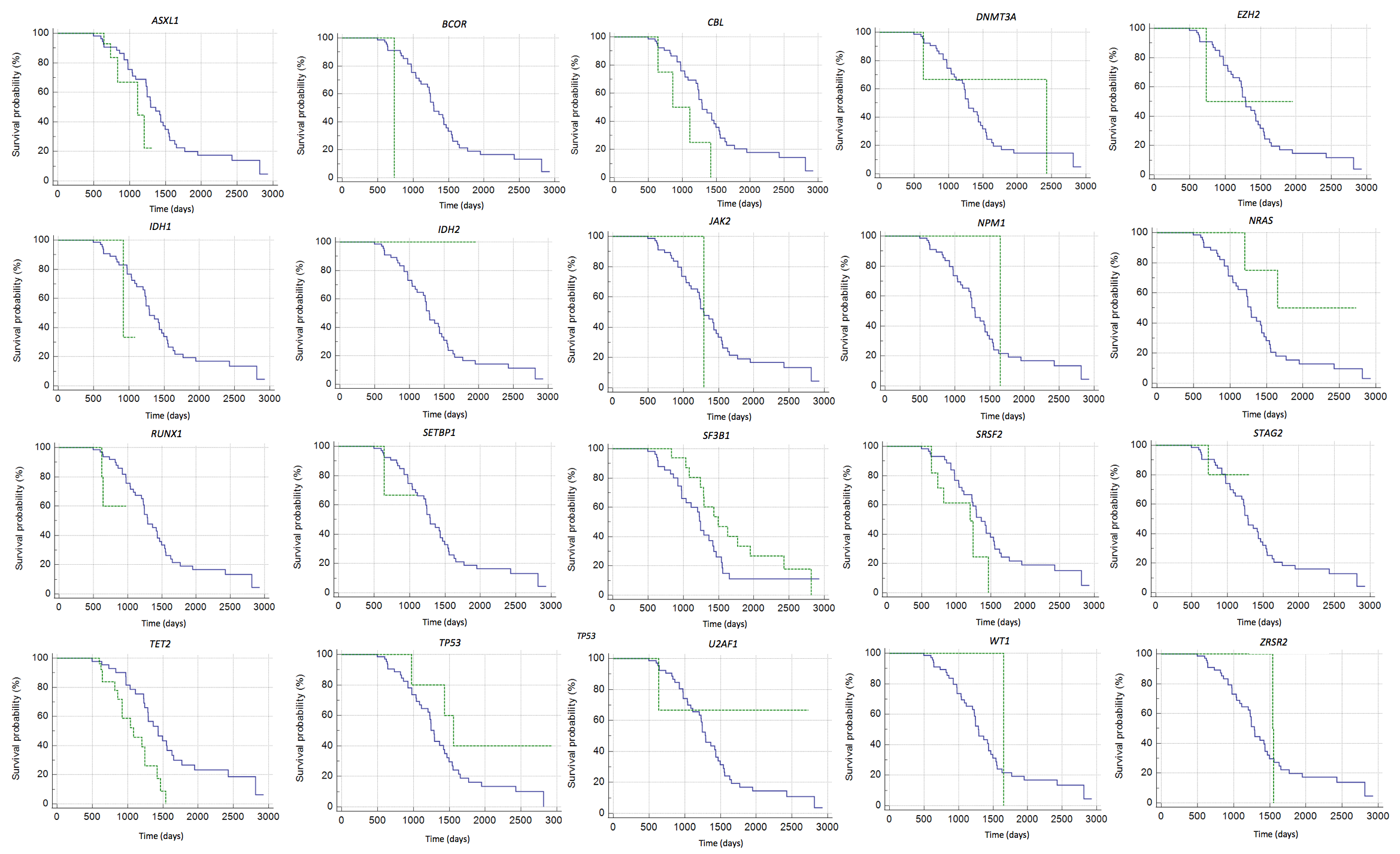

Supplement: Supplementary Figure 2 — Survival curve analysis based on gene mutations affecting a specific pathway. [file Image_2.TIFF]

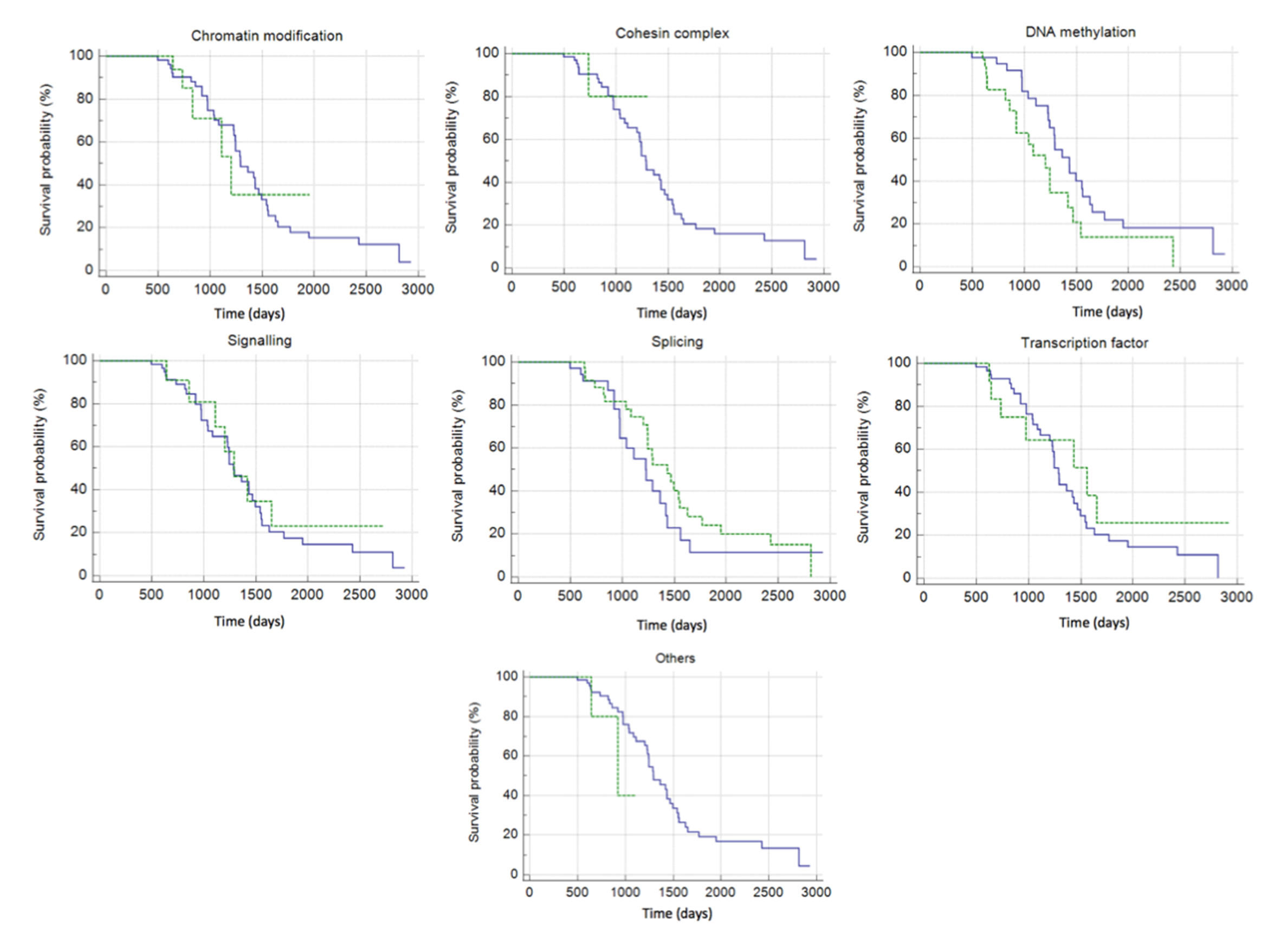

Supplement: Supplementary Figure 3 — Overall survival stratified according to risk class (blue: very poor, light green: poor, orange: intermediate, green: good and violet: very good). [file Image_3.TIFF]
