# Supplementary material for: Somatic Mutations and the Risk of Undifferentiated Autoinflammatory Disease in MDS: An Under-Recognized but Prognostically Important Complication
Source: Front Immunol. 2021 Feb 19;12:610019. doi: 10.3389/fimmu.2021.610019 (PMC7933213; doi:10.3389/fimmu.2021.610019)
Supplement: Supplementary Table 1 — Established diagnoses of autoinflammatory disease (AD) and additional inflammatory features. [file Table_1.DOCX]

**Table S1. Established diagnoses of autoinflammatory disease (AD) and additional inflammatory features**

| Patients | AD diagnosis | Atypical symptoms/signs for AD diagnosis |
| --- | --- | --- |
| AD1 | PMR | Persistent Inflammatory arthropathy |
| AD2 | Ulcerative colitis | None |
| AD3 | Ulcerative colitis | None |
| AD4 | Giant cell arteritis | None |
| AD5 | PMR | None |
| AD6 | Ulcerative colitis, PMR | None* |
| AD7 | PMR, gout | None* |
| AD8 | Giant cell arteritis | Nil |
| AD9 | SpA, Crohn's, Behcet's | Fevers* |
| AD10 | PMR | Fevers, inflammatory arthritis* |
| PMR-polymyalgia rheumatica; SpA-Spondyloarthritis | | |
| *disease resistant to standard treatment or needing prolonged course of corticosteroids | | |

**Table S2. Multivariate logistic regression analysis of the determinants associated with overall auto-inflammation.**

| **Variables** | **OR** | **95%CI** | **Statistical significance** |
| --- | --- | --- | --- |
| Age | 1.05 | 0.99-1.12 | NS |
| Sex | 1.31 | 0.44-3.88 | NS |
| Transcription factors pathway | 0.31 | 0.08-1.30 | NS |
| Abnormal karyotype | 1.45 | 0.12-17.67 | NS |
| Risk class |  |  |  |
| Poor vs very poor | 0.28 | 0.02-3.93 | NS |
| Intermediate vs very poor | 0.62 | 0.07-5.34 | NS |
| Good and very good vs very poor | 0.50 | 0.03-8.44 | NS |
| Treatment |  |  |  |
| Chemotherapy | 10.23 | 0.80-131.36 | NS |
| G-CSF | 0.47 | 0.12-1.94 | NS |
| Bone-marrow transplant | 5.88 | 0.41-84.70 | NS |
| Hypomethylating agents | 2.81 | 0.81-9.69 | NS |
| Hydroxyurea | 0.21 | 0.01-4.80 | NS |

**Table S3.** **Multivariate logistic regression analysis of the determinants associated with undifferentiated autoinflammation.**

| **Variables** | **OR** | **95%CI** | **Statistical significance** |
| --- | --- | --- | --- |
| Age | 1.00 | 0.95-1.05 | NS |
| Sex | 1.89 | 0.68-5.26 | NS |
| Transcription factors pathway | 0.69 | 0.20-2.31 | NS |
| Abnormal karyotype | 0.55 | 0.07-4.60 | NS |
| Risk class |  |  |  |
| Poor vs very poor | 1.46 | 0.09-24.02 | NS |
| Intermediate vs very poor | 3.10 | 0.40-24.23 | NS |
| Good and very good vs very poor | 2.18 | 0.17-28.16 | NS |
| Treatment |  |  |  |
| Chemotherapy | 1.46 | 0.12-17.47 | NS |
| G-CSF | 0.50 | 0.17-1.52 | NS |
| Bone-marrow transplant | 0.85 | 0.07-10.34 | NS |
| Hypomethylating agents | 0.32 | 0.09-1.07 | NS |
| Hydroxyurea | 0.12 | 0.00-3.67 | NS |

**Table S4.** **Multivariate logistic regression analysis of the determinants associated with well-defined autoinflammation.**

| **Variables** | **OR** | **95%CI** | **Statistical significance** |
| --- | --- | --- | --- |
| Age | 1.01 | 0.91-1.13 | NS |
| Sex | 2.28 | 0.27-19.44 | NS |
| Transcription factors pathway | 0.89 | 0.05-16.10 | NS |
| Risk class |  |  |  |
| Poor vs very poor | 0.28 | 0.01-15.25 | NS |
| Intermediate, good and very good vs very poor | 5.53 | 0.12-254.28 | NS |
| Treatment | 0.69 | 0.02-31.72 | NS |
